# Hemicellulose Recovery from Spent-Sulfite-Liquor: Lignin Removal by Adsorption to Resins for Improvement of the Ultrafiltration Process

**DOI:** 10.3390/molecules25153435

**Published:** 2020-07-28

**Authors:** Basel Al-Rudainy, Mats Galbe, Ola Wallberg

**Affiliations:** Department of Chemical Engineering, Lund University, SE-221 00 Lund, Sweden; mats.galbe@chemeng.lth.se (M.G.); ola.wallberg@chemeng.lth.se (O.W.)

**Keywords:** galactoglucomannan, lignin, lignin–carbohydrate-complex, lignosulfonates, adsorption, ultrafiltration, spent-sulfite-liquor

## Abstract

In this work, three polymeric resins were examined as alternatives for the separation of hemicellulose and lignin. The aim was to remove the lignin from spent-sulfite-liquor (SSL) prior to ultrafiltration, producing a hemicellulose-rich retentate with high purity, and increase the capacity of the membrane filtration. The lignin in the SSL was sulfonated; thus, two of the resins were anion exchangers and 1 was hydrophobic. The data from the equilibrium studies and adsorption kinetics were fitted to established models, and the results were interpreted based on these observations. The strongly basic anion exchanger performed best with regard to lignin removal. The adsorption followed the Sips isotherm, indicating that the process was cooperative with chemisorption as the main reaction between the adsorbate and adsorbent based on the kinetics. Regeneration of the adsorbent was also possible, wherein 100 g/L NaCl was sufficient to recover 98% of the lignin. The lignin removal had a positive effect on the ultrafiltration process, in which the flux increased by 38% and the extent of separation between the hemicellulose and lignin rose from 17% to 59%.

## 1. Introduction

There is growing interest in replacing fossil-based carbon sources with renewable bio-based alternatives. To this end, forest-based lignocellulosic biomass is a promising renewable alternative. It contains primarily cellulose, hemicellulose, and lignin of which cellulose is the most widely known and utilized component. Because lignin and hemicellulose account for over 50% of the available biomass, greater utilization of this material is important with regard to sustainability [1]. In the past several decades, lignin has been the focus regarding the development of various extraction methods and applications [2,3]. This strategy has led to the development of products such as adhesives, plasticizers, and biofuels [4,5,6].

Conversely, hemicellulose is a promising raw material for the production of barrier films, hydrogels, surfactants, and biofuels [7,8,9,10]. However, unlike lignin, large-scale production of hemicellulose does not exist, impeding the development of applications and the commercialization of hemicellulose-based products [11]. This deficiency has rendered research on the production of hemicellulose important for the advancement of sustainability goals.

Hemicellulose can be extracted from wood using such techniques as pressurized hot-water extraction, microwave heat fractionation, and steam explosion [12,13,14]. However, regardless of technique, all of them require the design of an entire process, which also entails separation and recovery steps. An alternative source from which hemicellulose can be recovered is waste streams in the pulp and paper industries, which remain largely untapped [15]. By utilizing the waste streams in pulp mills, their product portfolio will expand, and the load on the wastewater treatment plant will decrease.

Spent-sulfite-liquor (SSL) is a waste stream that is generated during the production of sulfite pulp. SSL is usually concentrated using evaporators and incinerated to recover pulping chemicals and generate power and heat [16]. SSL from conventional pulp and paper industries usually contains monosaccharides, lignosulfonates, extractives, and pulping chemicals [17]. When the pulping is performed in two stages, one of which has a pH of 4 to 5, the SSL also contains polysaccharides, such as hemicelluloses, but also lignin–carbohydrate complexes (LCCs) [11,17]. In this work, SSL was taken from the first step of the 2-step sodium-based sulfite pulping of spruce and pine, in which polymeric hemicellulose exists and is intact. SSL from spruce and pine contains primarily the hemicellulose galactoglucomannan, as expected in softwoods. Other minor hemicelluloses in SSL are xylan-based, but arabinogalactans are also present [18,19]. Given this complex mixture of polysaccharides, monosaccharides, lignosulfonates, and their complexes, its separation is challenging.

Membrane filtration technology has been used to separate similar waste streams, generating promising results [20,21,22]. Persson and Jönsson [20] concentrated a solution (softwood masonite wastewater) that contained 1–2 g/L to 60 g/L hemicellulose, reducing the volume by 99%. Hydrophobic compounds, such as lignin, were separated prior to the membrane filtration using activated carbon. The authors concluded that this method was an effective solution, but the cost of the activated carbon was high, and the regeneration was difficult. Krawczyk, et al. [22] combined membrane filtration and enzymatic treatment to separate high-molecular-weight hemicelluloses from chemi-thermomechanical process water. The group separated impurities such as colloids and fibers by microfiltration and concentrated the microfiltration permeate using ultrafiltration. The retentate was enzymatically treated to polymerize the hemicellulose–lignin complexes and further ultrafiltered to remove smaller, unreacted polysaccharides. The process yielded a high-quality hemicellulose solution with a concentration of 54 g/L.

Membrane filtration alone can be used in certain cases to separate and purify hemicellulose solutions [23,24]. However, in many cases, the combination of membrane filtration with another separation technique or pretreatment of the raw material is needed [18,23], perhaps because the hemicellulose and lignin have a slight difference in molecular weight, there are other impurities that hinder the separation, fouling increases the retention of all solutes, or they exist as LCCs [18,23].

As discussed above, Persson and Jönsson [20] and Krawczyk, et al. [22] presented several solutions for these problems. The use of activated carbon to remove hydrophobic compounds, such as lignin, increases the membrane filtration capacity (i.e., decreases fouling) and separates compounds. However, given the difficult task of regenerating activated carbon and recovering the adsorbed lignin and due to the high price of the adsorbent, this was not feasible [20,25]. Krawczyk, et al. [22] solved the separation issue by changing the molecular weight of the hemicellulose enzymatically prior to the final ultrafiltration step. Although this method worked, it required the product to be altered and a membrane filtration step to be added. Anti-solvent precipitation has also been shown to be an effective solution for separation of hemicellulose and lignin using either alcohols or acetone [18,26,27].

In our previous study, membrane filtration prior to the precipitation step was beneficial for cases in which the solutions were diluted [18]. Using this method, a degree of separation (mass of galactoglucomannan (GGM) divided by the sum of the lignin and galactoglucomannan mass) and yields above 76% were achieved. Although this method is efficient with regard to separation, it requires additional high-energy-demanding processing steps to recover the anti-solvent to become a viable solution. In addition, ideally, the method should be used after membrane filtration, given that anti-solvent precipitation of dilute solutions requires large amounts of anti-solvent, and thus does not increase the membrane filtration capacity [18].

Polymeric resins have been used to separate lignin, lignosulfonates, and other phenolic compounds from many sources [28,29,30,31,32,33,34]. Nitzsche, et al. [29] implemented the hydrophobic resins XAD7HP and SEPABEADS SP700 to remove 100% of the lignin from beech-wood hydrolysate, with a hemicellulose recovery of over 92%. The adsorption of phenol followed the Freundlich equation in single-component experiments and the extended Freundlich isotherm in multicomponent experiments. Schwartz and Lawoko [30] used XAD-4 hydrophobic resin to remove acid-soluble lignin from acid-hydrolyzed hemicelluloses (hardwood). The resin removed 100% of the furan derivatives and 90% of the acid-soluble lignin.

Narron, et al. [33] applied XAD16N, removing approximately 90% of the soluble lignin hardwood autohydrolysate and determined by NMR that the sugar loss was due primarily to the existence of LCCs. Heinonen, et al. [34] used the same type of resin on monosaccharide-rich hardwood hydrolysates and removed 80% of the lignin, with minimal loss of sugar (95% recovery). The primary goal of most of these studies was to produce high-quality lignin or improve the downstream fermentation process. Koivula, et al. [28] aimed to recover hemicellulose from birch and pine/eucalyptus hydrolysates and simultaneously enhance the membrane filtration by adsorbing the membrane foulants onto XAD-7 and XAD-16, decreasing membrane fouling and improving the flux. They also found that the decline in fouling was due to the removal of lignin-like material from the hydrolysates and that with large amounts of adsorbent, the loss of hemicellulose was significant. The type of lignin in these studies was not reported to contain sulfonic acid groups and was thus not comparable with the SSL in our work.

However, a similar resin (XAD-7) was used by Sumerskii, et al. [31] to isolate lignosulfonates from SSL, with which a lignosulfonate with high purity was generated, and the method was reported to be faster than ultrafiltration. The lignosulfonates that were produced using this method contained lower levels of sulfonic acid groups, which the group attributed to the high hydrophilicity of the lignosulfonates and the desorption that occurs during the washing of the resin.

Charged polymeric resins, or ion exchangers, constitute a potentially significant separation method. Lignosulfonates are negatively charged; thus, anion exchangers could be used to selectively adsorb the lignosulfonates. A patent by Van Blaricom and Russell [35] describes a method for separating lignosulfonates from ammonium-based SSL using anion exchange resins. According to these authors, a resin that contains primary or secondary amino groups is unsuitable for lignosulfonate-containing solutions, because such groups react with the lignosulfonates or other groups in the SSL and thus decrease the adsorption capacity. They suggested using resins with tertiary amino groups, which yield weakly basic resins, instead of quaternary amino groups, which generate strongly basic resins. The authors found that strongly basic resins were less efficient in the regeneration using sodium hydroxide. The authors used weakly basic resins to adsorb lignosulfonates from SSL (pH of approximately 4) and observed no adsorption of monosugars. The effluent after the adsorption had a pH of 9 to 11 when the free base form of the resin was used, which did not pose a problem, because the effluent contained ammonium hydroxide, which could be recovered and used in the pulping process.

Takahashi, et al. [36] removed acetic acid from SSL prior to fermentation to mitigate the inhibition of ethanol production using a strong-base anion-exchange resin to adsorb acetic acid. However, lignosulfonates were also adsorbed and thus decreased the amount of acetic acid that could be adsorbed. The (OH^−^) form of the resin also created problems, because the pH of the solution changed, in turn decomposing the monosugars in the solution. These problems were addressed by performing the adsorption in two stages with a neutralization treatment using CaO and CO_2_, resulting in 90% removal of acetic acid and greater ethanol production.

Liu, et al. [37] used a fixed-bed column with weakly basic anion exchangers to separate magnesium lignosulfonates from xylo-oligosaccharides, obtaining adsorption capacities as high as 390 mg/g resin and recovery yields of 98% for lignosulfonates and 93% for xylo-oligosaccharides. Other studies have performed lignosulfonate adsorption as a pretreatment method prior to further processing; thus, this method was not their focus [38,39].

The goal of this work was to remove lignin (here also lignosulfonates) from SSL using anion exchangers and hydrophobic resins. The adsorption data were fitted to commonly used adsorption models, and the results were interpreted based on the models and the underlying mechanisms. Another important factor of the industrial use of these resins is their regenerative ability. Thus, lignin desorption by the best-performing resin was also studied, and the SSL with the lower lignin content was filtered and compared with the original SSL solution.

## 2. Results and Discussion

### 2.1. Screening of Adsorbents

The raw material in this study was sodium-based SSL with a pH of approximately 4.5, containing primarily lignin (including lignosulfonates), pulping chemicals, and polysaccharides, as seen in Table 1. GGM was the main hemicellulose that was detected, with a monomeric composition of 0.41:0.36:1 (galactose/glucose/mannose). These values are higher than what was expected for pure GGM [40], based on our previous report [18], due to the presence of other galactan-based polysaccharides, such as arabinogalactan and β-galactan. Other minor hemicelluloses in the solution were xylan-type, as evidenced by the amounts of xylan and arabinan. Overall, this batch of SSL did not differ in composition from what has been reported [11,18,23,41].

Three resins were used to screen adsorbents: strong basic (IRA958), weak basic (IRA67), and hydrophobic (XAD4). The anion exchangers were chosen based on the functional group composition, for which a tertiary or quaternary amine was a requirement [35]. The hydrophobic resin was selected based on previous successful utilization of the resin in lignin removal applications [30].

**Figure 1:**
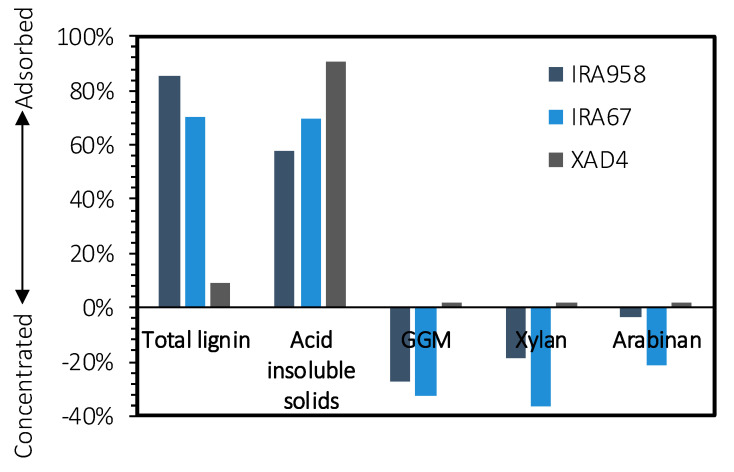
Removal of solutes from the SSL using the resins under examination. Negative removal indicates that the solute was concentrated in the solution.

The results of the adsorption screen for the raw SSL solution are presented in Figure 1. At first glance, the two adsorbents that performed best in selectively removing the lignin were IRA958 and XAD4. IRA958 outperformed all of the other adsorbents in adsorbing the highest amount of lignin per gram of adsorbent, resulting in a total removal of 85%. XAD4 poorly adsorbed the acid-soluble lignin (which included lignosulfonates) but removed acid-insoluble lignin well, with rates reaching as high as 90%. IRA958 and IRA67 removed a substantial amount of acid-insoluble lignin, suggesting that the acid-insoluble lignin was sulfonated before the acid hydrolysis, explaining the desulfonation and consequent precipitation of the lignin [42].

Coupling of the acid-insoluble lignin to the carbohydrates was also suggested by the adsorption of polysaccharides to XAD4, as seen in Figure 1. The amounts of polysaccharides adsorbed on the XAD4 was low, the polysaccharides together with the lignin were desorbed to confirm that the polysaccharides did indeed adsorb on the resin and that the results presented in Figure 1 were not due to errors. The XAD4 was rinsed with a total of 60 mL deionized water (20 mL for each rinse), and the adsorbates were desorbed sequentially using 60 mL 4 g/L sodium hydroxide solution and 60 mL methanol (20 mL for each rinse).

Through this procedure, 25% of the total adsorbed acid-insoluble lignin was recovered, of which 4.7% comprised polysaccharides. The composition of the desorbed polysaccharides with regard to xylan and arabinan was the same as in Table 1 but differed for GGM, which had a monomeric composition of 0.50:0.80:1 (Gal/Glu/Man). The comparison of desorbed GGM with the GGM in the raw material suggested that a major portion of the LCCs in this fraction comprised a bond between the glucose or mannose component of the GGM and lignin, possibly phenyl glycosidic bonds [23]. No adsorption of polysaccharide was observed in this initial screen for IRA958 and IRA67; instead, the concentration of polysaccharides increased. The adsorbents were dry before use, and according to the manufacturer, the moisture-retaining capacity of the resins ranges from 60% to 80%.

If the resins had swelled using the SSL as a wetting agent, the concentration of polysaccharides would have been the same in the bulk solution as in the resins themselves (assuming no adsorption of the solutes). However, the increase in polysaccharide concentrations suggests that not only did the resins swell, they also held back polysaccharides. The reason for these observations is unknown, but we hypothesize that the polysaccharides were larger than the pores on the resin, which led to the absorption of water molecules and the retention of large polysaccharides.

Based on these results, IRA958 was chosen for subsequent study, because it had the highest lignin removal per weight of adsorbent and no observable loss of polysaccharides. Another reason for this choice is that IRA958 (chloride form) did not raise the pH to a basic value, as IRA67 (free base) had done, increasing the pH to as high as 9 in certain samples, potentially deacetylating the GGM and degrading the polysaccharides [36].

### 2.2. Equilibrium Adsorption for IRA958

**Figure 2:**
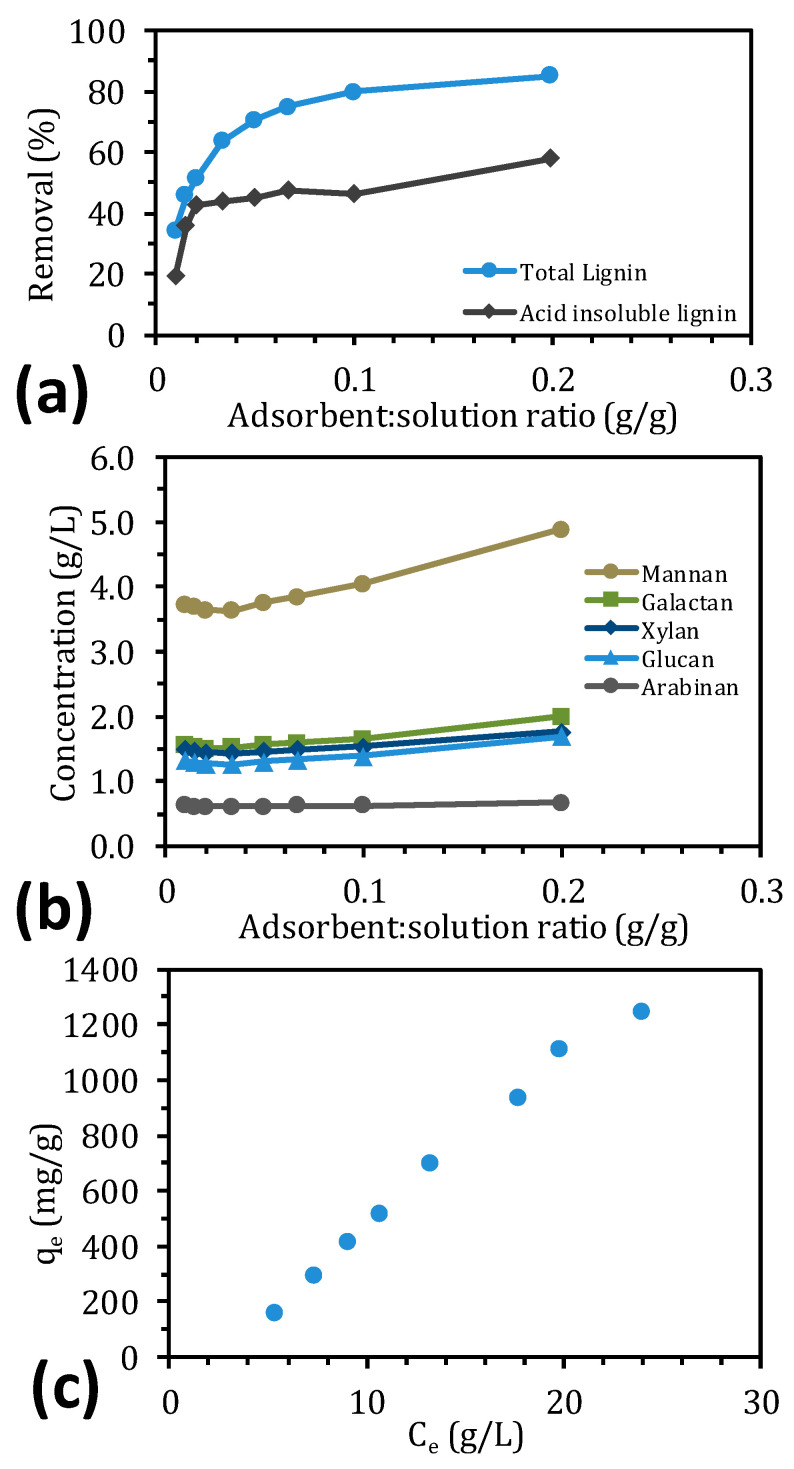
Lignin removal from solution (**a**) and the concentration of polysaccharides (**b**) at various adsorbent/solution ratios. (**c**) Amount of adsorbed lignin per gram of adsorbent (q_e_) vs. the concentration at equilibrium (C_e_).

In the screening study, a fixed adsorbent/solution ratio was used to identify the adsorbent that had the highest adsorption of lignin per weight of resin. To better understand the type of adsorption that occurred and determine the maximum adsorption capacity, an equilibrium adsorption study was performed in which the adsorbent/solution ratio was varied. With IRA958, lignin adsorption rose with increasing amounts of adsorbent (the amount of SSL solution was constant) (Figure 2a). The highest and most substantial removal of lignin occurred between 0 and 0.1 g adsorbent/g solution, wherein the removal reached as high as 80%. The remaining increase of approximately 5% required the addition of 0.1 g adsorbent/g solution, twice the amount that was needed to remove 80% of the lignin. The removal of acid-insoluble lignin was substantial with little adsorbent (approximately 42% removal) but nearly constant for the entire interval, increasing to 58% only at the end.

The adsorption of polysaccharides was not detected during the screening (Figure 1) but clearly showed a decrease of the concentration at low adsorption/solution ratios (up to 0.02 g/g), as seen in Figure 2b. At higher adsorbent/solution ratios (up to 0.1 g/g), the concentration of polysaccharides increased by approximately 8%, rising by another 21% with the last 0.1 g/g increment.

To describe the adsorption of lignin to IRA958, common adsorption isotherm models were used (Table 2). Data on the adsorptive capacity versus the lignin concentration were used to fit the models at equilibrium, as seen in Figure 2c. The data were not linear, especially near the higher concentration ranges, which explains the poor fit of the linear model (Table 2). Although the saturation did not plateau, the data at the high concentration indicate that the adsorption capacity leveled. The linear region at low concentrations does not intercept the y-axis (when extrapolated), indicating that the curve is S-shaped. The Langmuir model fails to describe these curves [43], which was also evident from the R^2^ value of 0.5445 and the negative coefficients, which have no tangible meaning (Table 2). The Freundlich isotherm fit these data better, and although the model is empirical, it has been used to describe multilayer adsorption systems on heterogeneous surfaces [37].The intensity of the adsorption (n_F_) was higher than unity, which indicates cooperative adsorption and that the curve has an S-shape [44,45]. Although R^2^ was close to unity, the model still failed to fit the data at high concentrations, which is a known limitation of this model [46]. The Sips and Brunauer–Emmett–Teller (BET) models have been used to overcome these issues. The BET equation is a multilayer adsorption model that has no limits in the number of adsorption layers that are available on the homogeneous surface of the adsorbent [43]. In addition, the BET model resolves a limited amount of S-shaped experimental data, which explains the low R^2^ value. Conversely, the Sips isotherm was derived to account for the limitations of the Freundlich and Langmuir models. When the concentration is low, the isotherm resembles the Freundlich model, versus the Langmuir model when the concentration is high [46]. The results in Table 2 show that the Sips model fit our data the best and that the exponent (n_S_) was larger than unity, indicating that the type of adsorption was cooperative, as seen with the Freundlich model. Further, the maximum adsorption capacity (Q_s_) was unable to be predicted using the Freundlich model and was calculated to be 1947 mg adsorbate/g adsorbent. The calculated maximum adsorption capacity was higher than in the study by Liu, et al. [37], perhaps due to differing lignosulfonate types, the molecular weight of the adsorbates, the type of adsorbent, and the fact that the resins were dry before adsorption.

### 2.3. Adsorption Kinetics

**Figure 3:**
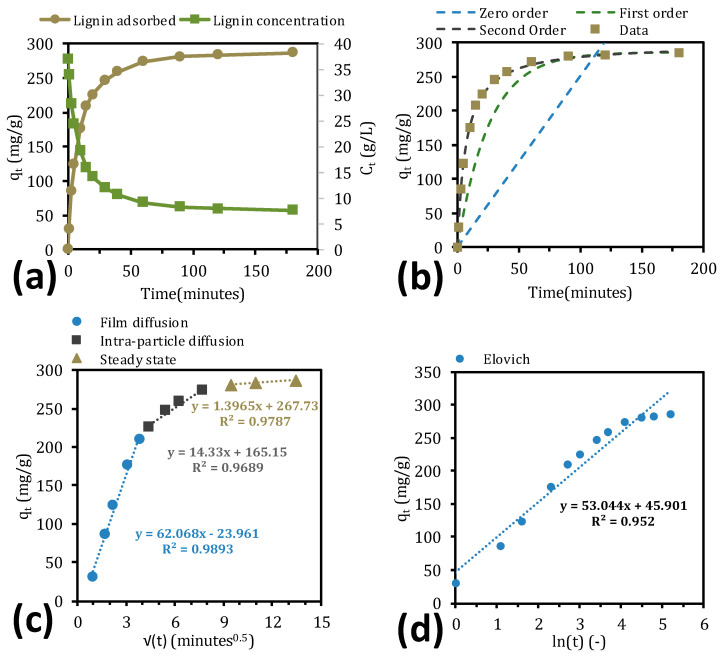
Lignin adsorption and concentration vs. time (**a**) and the resulting fit of the kinetic models to the data (**b**–**d**).

Adsorption kinetics are important when identifying the mechanisms of adsorption and can also be used for simulations and scale-up of the process [51]. The kinetic models that were chosen for the modeling were the commonly used pseudo zero-, first-, and second-order expressions but also the Elovich and intra-particle diffusion, as presented in Table 2. The data that were used for the models are shown in Figure 3a, in which the lignin that has adsorbed and the lignin concentration in the bulk are plotted versus time.

The trend was similar to what has been reported [37], the major difference being the time scale. In Figure 3a, the entire adsorption process took 2 to 3 h to reach equilibrium compared with the 6 to 8 h in the literature; as discussed in the previous section, the difference in solutes, adsorbents, and operating conditions could have resulted in this deviation. Between pseudo-kinetic models, the second-order equation gave the best fit (R^2^ = 0.9997), indicating that the adsorption type was chemisorption [51]. The calculated adsorption capacity at equilibrium (*q_e_*) was closer to what was observed experimentally (286.3 mg/g) for the pseudo-first-order equation (Table 2); however, this model did not properly resolve the kinetics in the region between *t* = 0 and 75 min (Figure 3b).

The intra-particle diffusion and Elovich models (Figure 3c,d) gave more information regarding the adsorption process. According to the Elovich model, the initial adsorption rate for the lignosulfonates was approximately 126 mg/(g min), which is 30 times higher than in the literature [37]. Moreover, according to the intra-particle diffusion model, the rapid adsorption of lignin at the outset was due primarily to film, boundary layer, or macropore diffusion, which occurred in the first 10 min of adsorption (Figure 3c). The subsequent slow adsorption (t > 10 min) was attributed to intra-particle diffusion, followed by a steady-state interval in which the intra-particle diffusion rate constant (k_p_) began to approach 0.

### 2.4. Lignin Desorption

**Figure 4:**
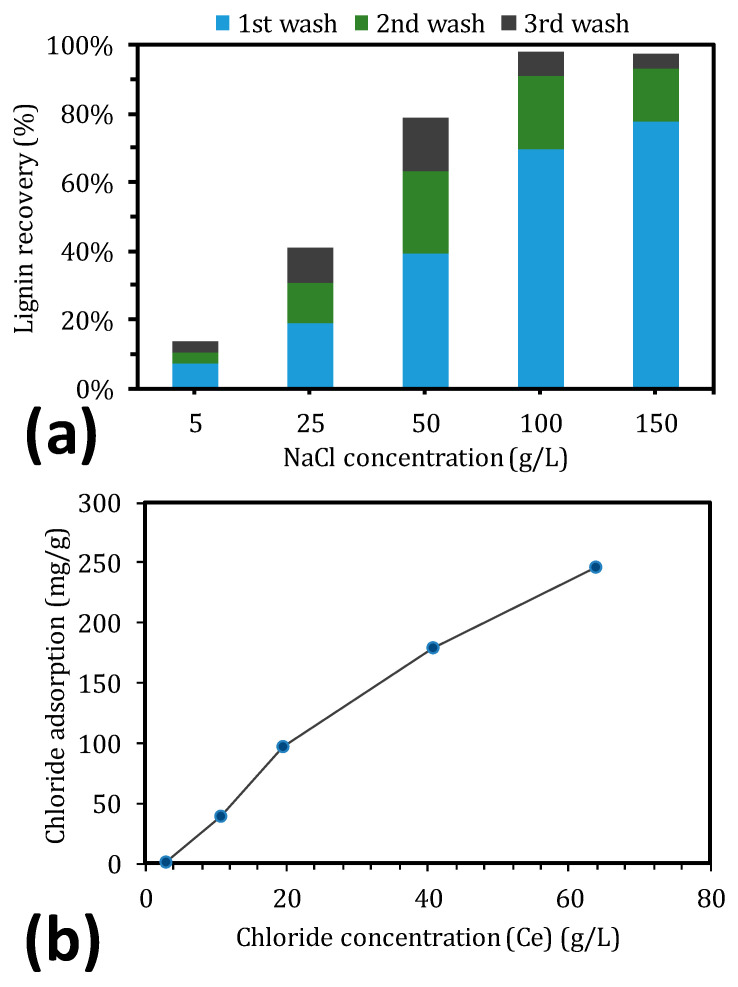
Lignin recovery vs. initial sodium chloride concentration at various wash steps (**a**). Chloride concentration at equilibrium during the first wash step (**b**).

For the implementation of the adsorption-type recovery process on an industrial scale, the recovery of lignosulfonates and the regeneration of the resin are important. These aspects were examined by washing the resin with various concentrations of the regenerant in a sequence of three washes (Figure 4). According to the results, 100 g/L sodium chloride was a suitable concentration of regenerant, effecting a total lignosulfonate recovery of 97.6% after three consecutive washes. Increasing the concentration further did not improve the desorption of lignosulfonates, as seen in Figure 4a. These results are similar to those of Liu, et al. [37], who obtained a lignosulfonate recovery of 98% using a regenerant concentration of 10% NaCl and 2% NaOH, with a total volume that was comparable with the one that we used. In addition, the purity of lignosulfonates was approximately 92%, which could be attributed to the salt that was used during the regeneration.

It is unknown whether the recovery of salts from the recovered lignosulfonates is beneficial for the process economy. Hilal, et al. [52] have shown that it is feasible to recover NaCl using nanofiltration membranes from highly concentrated salt solutions (up to 25,000 ppm), rendering the use of resins for the separation of lignosulfonates from GGM a promising industrial method.

According to the results in Figure 4, there remains the possibility to simplify the process by decreasing the number of washes that is required, as evidenced by the first wash, with which the lignin recovery clearly increased. The data in Figure 4b also indicate this pattern, because the saturation of chloride adsorption did not plateau. Using the Sips isotherm to fit the data in Figure 4b (R^2^ = 0.9972), a theoretical maximum adsorption capacity is reached at 364 mg chloride/g resin. However, to reach such a high level, the concentration of sodium chloride must approach the solubility limit, which is not desired, especially if the recovery of salts using nanofiltration is important [52,53].

### 2.5. Ultrafiltration before and after Adsorption

**Figure 5:**
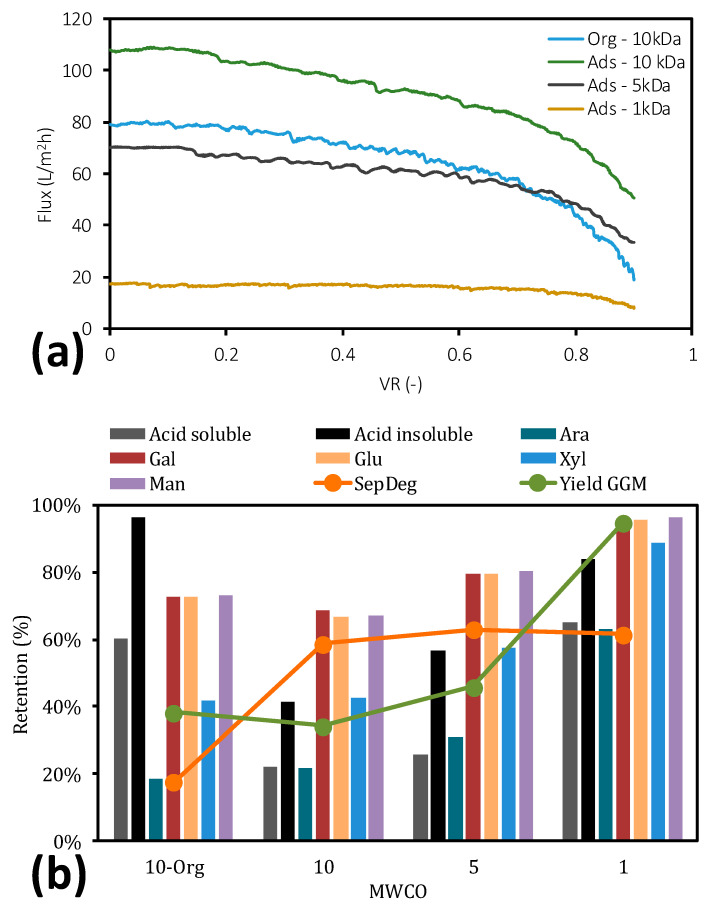
Flux vs. volume reduction (VR) during the ultrafiltration of treated and non-treated SSL (**a**). Retention of solutes during the ultrafiltration studies (**b**).

To determine the effects of adsorption on ultrafiltration, the delignified SSL was ultrafiltered and compared with the ultrafiltered raw SSL solution. The flux for the ultrafiltered raw SSL solution (Org − 10 kDa, Figure 5a) was 50% lower than that for the same solution and membrane in our previous study [23]. The retention of all solutes was also lower, likely due to the filtration temperature, room temperature in this study versus 50 °C in our earlier report. An increase in flux is usually followed by a decline in solute retention, and vice versa, as posited by the film theory [54]. However, in this case, we believe that the rise in temperature also softened the membrane, causing it to compress and thus lead to a decrease in pore size, in contrast to the higher retentions in our previous study [23,55].

The results in Figure 5a clearly show the effect of the adsorption prior to ultrafiltration (Ads – 10 kDa). The removal of lignin increased the flux by 38% (Org − 10 kDa vs. Ads − 10 kDa) as the degree of separation (Figure 5b) improved from 17% to 59%, which increased the capacity of the membrane filtration and the purity. The flux at the end of the membrane filtration (Ads − 10 kDa) was also higher than that for the untreated SSL solution, allowing the membrane filtration to proceed to a greater reduction in volume, resulting in higher concentrations and possibly higher purities (i.e., greater retention of polysaccharides compared with lignin) (Figure 5b). The retention of polysaccharides was not affected considerably by the lignin removal, given the minor change in mannose retention from 73% to 67%. This decline caused the GGM yield to decrease from 38% to 34%.

The reduction in yield can be compensated for by operating with a denser membrane. The results are presented in Figure 5 (Ads − 5kDa and Ads − 1 kDa). By switching to a 5-kDa cutoff membrane, the flux decreased compared with Ads − 10 kDa, as expected. However, the average flux was roughly the same as for the original solution (Org − 10 kDa), rendering this a promising alternative. The switch to a denser membrane also increased the retention of polysaccharides from 67% to 80%, which in turn improved the yield to 46%. The degree of separation rose from 59% to 63%, indicating that the treated SSL was purer than before. Incorporation of the densest membrane (1 − kDa cutoff) significantly increased the GGM yield (up to 95%). The degree of separation was approximately constant, indicating that the treated SSL did not increase in purity and that the lignin was concentrated, like the GGM, as noted for the retentions (Figure 5b). Although the densest membrane is promising, based on its high yield of GGM, the flux was four times lower than that of the original solution. Nevertheless, the flux was not 0, and whether the use of the densest membrane is economical requires a technoeconomic analysis.

## 3. Materials and Methods

### 3.1. Raw Material

The sodium-based SSL originated from the first step in a 2-step softwood (60% *Picea abies* and 40% *Pinus sylvestris*) sulfite pulping process (Domsjö Fabriker, Örnsköldsvik, Sweden).

### 3.2. Preparation of the Adsorbents

The adsorbents were produced by Sigma-Aldrich (Saint Louis, MO, USA) and consisted of a strong-base anion exchanger (Amberlite IRA958, chloride form), weak-base anion exchanger (Amberlite IRA-67, free base), and hydrophobic polyaromatic resin (Amberlite XAD4, 20–60 mesh). The resins were first soaked in deionized water (6 parts water to 1 part resin) for 3 h. The wash was repeated until the conductivity of the solution was less than 1 µS/cm, which required three washes for each resin. The washed resins were then dried in an oven at 50 °C for 48 h before the experiments.

### 3.3. Adsorbent Screen and Equilibrium Adsorption Studies

The screen was performed by adding excess dried adsorbent (4 g) to 50 mL vials that were filled with 20 mL of SSL solution, yielding a solution/adsorbent ratio of 5. The vials were mixed (continuous rotation on setting 2) in an incubator (combi-H12, FINEPCR, Gyeonggi-do, South Korea) at room temperature for 24 h. The adsorbent and remaining solution were carefully separated using a pipette before the solution was analyzed for lignin and carbohydrate composition.

The equilibrium studies were conducted by varying the amount of adsorbent that was added to the 50 mL vials: 0.20, 0.30, 0.40, 0.67, 1.0, 1.33, 2.00, and 4.00 g. The same amount of SSL and method were used as described for the screen. The adsorption capacity (amount of adsorbed material per weight dry adsorbent (*q*_e_)) was calculated per Equation (1):

(1) $$q_{e}\;\left(\frac{mg}{g}\right)=\frac{\left(C_{0}-C_{e}\right)V}{W}$$

where *C*_0_ and *C_e_* are the initial and equilibrium concentrations of the measured substance, respectively; *V* is the volume of the solution; and *W* is the weight of the dry adsorbent. 

The removal of solutes (percentage) was calculated per Equation (2):

(2) $$Removal\;\left(\%\right)=100\frac{\left(C_{0}-C_{e}\right)}{C_{0}}$$

### 3.4. Adsorption Kinetics

The adsorption kinetics were analyzed at room temperature in a 1 L bottle with 50 g of dry adsorbent and 500 mL of SSL. The solution and adsorbent were mixed continuously using a magnetic stirrer at 100 rpm. Samples were initially withdrawn (1 mL) once every minute, increasing to once every 5, 10, 20, and 30 min, for a total of 3 h, after which the change in lignin concentration was minimal. The adsorption capacity and lignin removal were calculated per Equations (1) and (2), where the equilibrium concentration (C_e_) was replaced with the concentration at time (*t*).

### 3.5. Lignin Desorption

Five samples were prepared, containing 7 g of wet adsorbent (approximately 2 g of dry adsorbent) from the kinetics study. Twenty milliliters of regenerant was added to each sample with varying concentrations of sodium chloride (5, 25, 50, 100, and 150 g/L) and a constant concentration of sodium hydroxide (10 g/L) per the recommendations in the Amberlite specifications datasheet. Both chemicals were analytical-grade and obtained from Merck Millipore (Burlington, MA, USA). This procedure was repeated three times to desorb as much lignin as possible for the given concentrations.

### 3.6. Membrane Filtration

**Figure 6:**
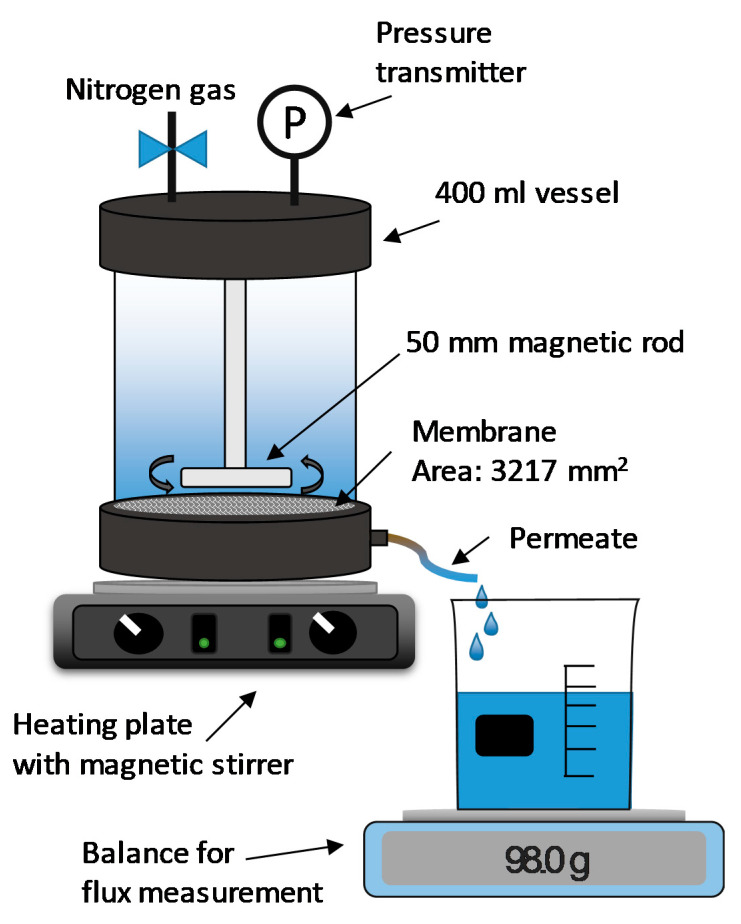
Illustration of the membrane filtration equipment.

The membrane filtration studies were performed using three flat-sheet regenerated cellulose membranes. The membranes had a molecular weight cutoff of 10 kDa (RC70PP, Alfa Laval Nordic A/S, Søborg, Danmark), 5 kDa (C5F, Microdyn-Nadir GmbH, Wiesbaden, Germany), and 1 kDa (Ultracel PLAC, EMD Millipore Co., Billerica, MA, USA). The equipment consisted of a 400 mL stirred module, as illustrated in Figure 6. The crossflow velocity was set by changing the rotational velocity of the magnetic rod [41], and the temperature was controlled using a heating plate (MR2002, Heidolph Instruments GmbH & Co.KG, Schwabach, Germany). The pressure was controlled using a valve that was connected to a nitrogen gas supply and monitored with a digital gauge (DCS40.0AR, Trafag AG, Bubikon, Switzerland). The flux was monitored using a digital balance (PL6001-l, Mettler Toledo Inc., Columbus, OH, USA) that was connected to a computer.

### 3.7. Analytical Measurements

#### 3.7.1. Total Dry and Ash Content

Three milliliters of various samples were weighed in ceramic crucibles and dried in an oven (Heraeus, Heraeus Holding GmbH, Hanau, Germany) at 105 °C for 24 h. The dry samples were weighed again, and the total dry content was calculated. The dry samples were placed in a furnace (B150, Nabertherm GmbH, Lilienthal, Germany) and heated slowly from room temperature to 900 °C over 1 h, after which they were left at 900 °C for 12 h. The samples were then weighed to determine the ash content.

#### 3.7.2. Hemicellulose and Acid-Insoluble Solids

The hemicellulose content and amount of acid-insoluble solids were measured according to previous studies [23]. Ten milliliters of the liquid samples was mixed with 750 µL 72% sulfuric acid and autoclaved (Systec DX 150, Wettenberg, Germany) at 121 °C for 1 h to hydrolyze the polymeric sugars. The samples were then filtered to remove insoluble solids, and the filters were dried overnight in an oven at 105 °C. The acid-insoluble solids content was determined by measuring the dried filters before and after filtration of the samples.

The hemicellulose content was determined by analyzing the liquid fraction after the filtration by high-performance anion-exchange chromatography (HPAEC). The HPAEC system (ICS-5000+ DC, Dionex, Sunnyvale, CA, USA) was a pulsed amperometric detector with a compartment temperature of 30 °C, and the separation was performed on a Carbo Pac PA1 analytical column. Deionized water at 1 mL/min was used as the main eluent, and 200 mM sodium hydroxide solution was used as the postcolumn addition at 0.5 mL/min. The samples were diluted with deionized water, and the injection volume for the HPAEC was 10 µL for all samples. The standards were D-mannose, D-xylose, D-glucose, D-galactose, and L-arabinose (Fluka Chemie AG, Buchs, Switzerland), and the hemicellulose content was determined after anhydro corrections of 0.90 for hexoses and 0.88 for pentoses.

#### 3.7.3. Lignin and Chloride Content

The lignin content in the samples was determined on a spectrophotometer (Shimadzu UV-1800, Kyoto, Japan) at a wavelength of 280 nm using an extinction coefficient of 13.01 L/(g cm). The chloride content was measured using titrator strips (Quantab Chloride Low Range titrators, HACH, Loveland, CO, USA).

## 4. Conclusions

The aim of this work was to remove lignin from SSL using two anion exchangers and one hydrophobic resin. The strong-base anion exchange resin (IRA958) performed best during the screen, obtaining the highest removal of total lignin. XAD4 (hydrophobic resin) removed the most acid-insoluble lignin, approximately 5% of which constituted polysaccharides. This finding suggests that the acid-insoluble lignin was bound to carbohydrates. Polysaccharides were not removed with the anion exchangers because the concentration of polysaccharides increased in the treated SSL. The equilibrium study with the best-performing resin (IRA958) showed that the S-shaped adsorption capacity–concentration curve followed the Sips isotherm, indicating that the adsorption was cooperative. The kinetics study with the same resin demonstrated that the adsorption was chemisorptive, reflected by film diffusion in the first stage of the adsorption and intra-particle diffusion for the remainder before reaching the steady state. The regeneration of adsorbent was possible using sodium chloride and sodium hydroxide, for which a concentration of 100 g/L was sufficient. Higher regenerant concentrations did not increase the total recovery of lignosulfonates, but they decreased the number of washes for regeneration that was required.

The removal of lignin from the SSL resulted in a 38% increase in ultrafiltration flux and a rise in separation degree from 17% to 59%. The GGM yield fell slightly during the ultrafiltration of the treated SSL as a result of the low retention compared with the nontreated SSL. It was possible, however, to increase the yield by using a membrane with a lower molecular-weight cutoff.

## Figures and Tables

**Table 1:** Composition of the spent-sulfite-liquor (SSL) raw material.

	Concentration (g/L)
Total dry substance	84.7
Ash	31.3
Acid soluble lignin	34.1
Acid insoluble lignin	2.0
Arabinan	0.6
Galactan	1.6
Glucan	1.4
Xylan	1.5
Mannan	3.9

**Table 2:** Adsorption models for fitting the lignin adsorption data. The equilibration concentration (*C_e_*) was expressed in mg/mL, the adsorption capacity (*q_e_* and *q_t_*) was expressed in mg/g, and time (*t*) was expressed in minutes.

Model	Fitted Parameters	Unit	R^2^	Equation	Ref
Adsorption isotherms					
Linear	K_lin_ = 51.67	(mL/g)	0.9679	$q_{e}=K_{lin}C_{e}$	[47]
Langmuir	Q_L_ = −1621.8 K_L_ = −0.0202	(mg/g) (mL/mg)	0.5445	$q_{e}=\frac{Q_{L}K_{L}C_{e}}{1+K_{L}C_{e}}$	[48]
Freundlich	K_F_ = 31.06 n_F_ = 1.1774	(mL^nF^mg^1−nF^/g) (-)	0.9855	$q_{e}=K_{F}C_{e}^{n_{F}}$	[48]
Sips (Langmuir–Freundlich)	Q_S_ = 1947.2 K_S_ = 0.0035 n_S_ = 1.9616	(mg/g) (mL/mg) (-)	0.9978	$q_{e}=\frac{Q_{S}K_{S}C_{e}^{n_{S}}}{1+K_{S}C_{e}^{n_{S}}}$	[43]
Modified Brunauer–Emmett–Teller (BET)	Q_B_ = 24,728 K_B_ = 0.3926 C_S_ = 216.74	(mg/g) (mL/mg) (mg/mL)	0.9839	$q_{e}=\frac{Q_{B}K_{B}C_{e}}{\left(C_{S}-C_{e}\right)\left(1+\left(K_{B}-1\right)\left(C_{e}/C_{s}\right)\right)}$	[49]
Adsorption kinetics					
Pseudo-zero-order	k_0_ = 2.5145	(mg/(g min))	−0.6910	$q_{t}=k_{0}t$	[50]
Pseudo-first-order	k_1_ = 0.0389 q_e_ = 286.44	(1/min) (mg/g)	0.9781	$\ln\left(q_{e}-q_{t}\right)=\ln\left(q_{e}\right)-k_{1}t$	[50]
Pseudo-second-order	k_2_ = 4.869e−4 q_e_ = 299.22	(g/(mg·min)) (mg/g)	0.9997	$\frac{1}{\left(q_{e}-q_{t}\right)}=\frac{1}{q_{e}}+k_{2}t$	[48]
Elovich	α = 126.02 β = 0.0189	(mg/(g·min)) (g/mg)	0.9520	$q_{t}=\frac{1}{\beta }\ln\left(\alpha \beta \right)+\frac{1}{\beta }\ln\left(t\right)$	[48]
Intra-particle diffusion	k_p_ = 14.33 m = 165.15	(mg/(g·min^0.5^)) (mg/g)	0.9689	$q_{t}=k_{p}\sqrt{\left(t\right)}+m$	[48]
